# An ortho­rhom­bic polymorph of the ultraphosphate YP_5_O_14_

**DOI:** 10.1107/S1600536809007193

**Published:** 2009-03-06

**Authors:** Aïcha Mbarek, Abdelghani Oudahmane, Malika El-Ghozzi, Daniel Avignant

**Affiliations:** a Laboratoire de Chimie Industrielle, Département de Génie des Matériaux, Ecole Nationale d’Ingénieurs de Sfax, Université de Sfax, BP W 3038, Sfax, Tunisia; b Laboratoire de Chimie du Solide Minéral, Département de Chimie, Faculté des Sciences Semlalia, Université Cadi Ayyad, Marrakech, Morocco; c Laboratoire des Matériaux Inorganiques, UMR CNRS 6002, Université Blaise Pascal, 24 Avenue des Landais, 63177 Aubière, France

## Abstract

Single crystals of yttrium penta­phosphate(V), YP_5_O_14_, were obtained by solid-state reaction. The ortho­rhom­bic title compound belongs to the family of ultraphosphates and is the second polymorph of this composition. It is isotypic with its Ho and Er analogues. The structure contains two bridging *Q* ^2^-type PO_4_ tetra­hedra and one branching *Q* ^3^-type PO_4_ tetra­hedron, leading to infinite ultraphosphate ribbons running along the *a* axis. The coordination polyhedron around the Y^3+^ cation may be described as distorted bicapped trigonal-prismatic. The YO_8_ polyhedra are isolated from each other. They are linked by corner-sharing to the O atoms of six *Q* ^2^-type and of two *Q* ^3^-type PO_4_ tetra­hedra into a three-dimensional framework.

## Related literature

Besides crystals of the title compound, crystals of the monoclinic polymorph were also obtained (Mbarek *et al.*, 2009 ▶). For isotypic structures, see: Durif (1972 ▶) for the Ho member; Katrusiak & Kaczmarek (1995 ▶) and Dimitrova *et al.* (2004 ▶) for the Er member. For a review of the crystal chemistry of ultraphosphates, see: Durif (1995 ▶). For applications of rare earth ultraphosphates, see: Rao & Devine (2000 ▶); Moine & Bizarri (2006 ▶). For general background, see: Porai-Koshits & Aslanov (1972 ▶).

## Experimental

### 

#### Crystal data

YP_5_O_14_
*M* *_r_* = 467.76
Orthorhombic, 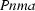

*a* = 8.7128 (2) Å
*b* = 12.7218 (4) Å
*c* = 8.9377 (3) Å
*V* = 990.68 (5) Å^3^
*Z* = 4
Mo *K*α radiation
μ = 6.79 mm^−1^
*T* = 296 K
0.22 × 0.15 × 0.11 mm

#### Data collection

Bruker APEXII CCD area-detector diffractometer
Absorption correction: multi-scan (*SADABS*; Sheldrick, 2008*a* ▶) *T*_min_ = 0.330, *T*_max_ = 0.460
5338 measured reflections
1194 independent reflections
1136 reflections with *I* > 2σ(*I*)
*R*_int_ = 0.019

#### Refinement

*R*[*F* ^2^ > 2σ(*F* ^2^)] = 0.030
*wR*(*F* ^2^) = 0.110
*S* = 1.22
1194 reflections
97 parameters
Δρ_max_ = 1.60 e Å^−3^
Δρ_min_ = −1.11 e Å^−3^

### 

Data collection: *APEX2* (Bruker, 2008 ▶); cell refinement: *SAINT* (Bruker, 2008 ▶); data reduction: *SAINT*; program(s) used to solve structure: *SHELXS97* (Sheldrick, 2008*b* ▶); program(s) used to refine structure: *SHELXL97* (Sheldrick, 2008*b* ▶); molecular graphics: *CaRIne* (Boudias & Monceau, 1998 ▶); software used to prepare material for publication: *SHELXTL* (Sheldrick, 2008*b* ▶).

## Supplementary Material

Crystal structure: contains datablocks global, I. DOI: 10.1107/S1600536809007193/wm2221sup1.cif

Structure factors: contains datablocks I. DOI: 10.1107/S1600536809007193/wm2221Isup2.hkl

Additional supplementary materials:  crystallographic information; 3D view; checkCIF report

## Figures and Tables

**Table 1:** Selected bond lengths (Å)

Y1—O6	2.278 (3)
Y1—O6^i^	2.278 (3)
Y1—O4^i^	2.341 (3)
Y1—O4	2.341 (3)
Y1—O8^i^	2.391 (4)
Y1—O8	2.391 (4)
Y1—O1	2.455 (5)
Y1—O3	2.518 (5)
P1—O8^ii^	1.455 (4)
P1—O2	1.549 (4)
P1—O5	1.553 (3)
P1—O7	1.567 (3)
P2—O3^iii^	1.460 (5)
P2—O1	1.479 (5)
P2—O2	1.623 (4)
P2—O2^i^	1.623 (4)
P3—O4	1.467 (3)
P3—O6^iv^	1.468 (3)
P3—O7^v^	1.607 (3)
P3—O5^vi^	1.611 (3)

Symmetry codes: (i) 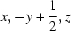; (ii) 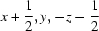; (iii) 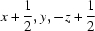; (iv) 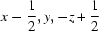; (v) 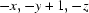; (vi) 
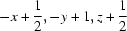
.

**Fig. 1.**
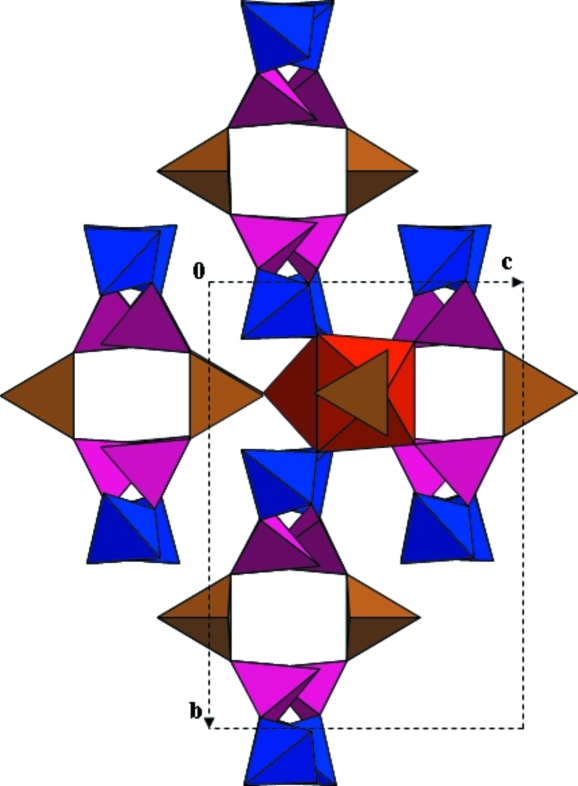
Projection along [100] of the structure of YP5O14 showing the isolated ∞(P5O14)3- ribbons.

**Fig. 2.**
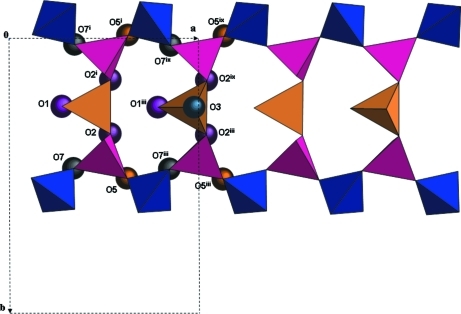
Details of the ∞(P5O14)3- ribbon in a projection along [001], symmetry codes: (i) x, -y+1/2, z; (iii) x+1/2, y, -z+1/2; (ix) x+1/2, 1/2-y+1/2, 1/2-z.

## supplementary crystallographic information

### Comment

Rare earths ultraphosphates exhibit a growing attention because of their potential applications as optical materials, including lasers, phosphors, matrices for the energy up-conversion and more recently for plasma display panels (PDP) as they exhibit an absorption band overlapping the emission spectrum of a Xe—Ne plasma in the VUV region (Rao & Devine, 2000; Moine & Bizarri, 2006). The non-optically active matrix of YP_5_O_14_ can be used as host material for such applications and hence needs to be structurally well-characterized. This article deals with the crystal structure refinement of the orthorhombic polymorph that is isotypic with HoP_5_O_14 _ (Durif, 1972) and ErP_5_O_14_ (Katrusiak & Kaczmarek, 1995; Dimitrova *et al.* 2004).

Two PO_4_ tetrahedra are Q^2^ type bridging tetrahedra with typical two shorter and two longer P—O bonds (Durif, 1995). The third PO_4_ tetrahedron is a branching Q^3^ type tetrahedron and exhibits also characteristic bond lengths ranging from 1.455 (4) to 1.567 (3) Å. These PO_4_ groups form infinite ribbons with composition (P_5_O_14_)^3-^ which can be considered as built of two infinite (PO_3_)_n_ chains running along the *a* axis and connected by alternating up and down capping PO_4_ tetrahedra (Figs. 1, 2). The repetition unit in these ribbons is P_10_O_28_.

The coordination polyhedron around the Y^3+^ cation is a distorted bicapped trigonal prism according to criteria defined by Porai-Koshits & Aslanov (1972) with (δ_1_ = 0°, δ_2_ = 18.28°, δ_3_ = δ_4_ = 42.87°; theoretical values δ_1_ = 0°, δ_2_ = 21.7°, δ_3_ = δ_4_ = 48.2°). The YO_8_ polyhedra are isolated from each other. They are linked by corner-sharing to six Q^2^ type PO_4_ tetrahedra and to two Q^3^ type tetrahedra leading to the three-dimensional framework.

### Experimental

Crystals of the title compounds were synthesized by reacting Y_2_O_3_ with (NH_4_)_2_HPO_4_ in a graphite crucible. A mixture of these reagents in the molar ratio 1:9 was used for the synthesis. The mixture was first heated at 473 K for 12 h. Then the temperature was raised up to 673 K and was held for 2 days before cooling to room temperature at a rate of 10 K/h. Single-crystals were extracted from the batch by washing with hot water. Besides crystals of the title compound, crystals of the monoclinic polymorph were also obtained (Mbarek *et al.*, 2009).

### Refinement

The highest residual peak in the final difference Fourier maps was located 0.26 Å from atom Y1 and the deepest hole was located 0.44 Å from atom P2.

### Crystal data

**Table:**

YP_5_O_14_	*F*(000) = 904
*M**_r_* = 467.76	*D*_x_ = 3.136 Mg m^−^^3^
Orthorhombic, *P**n**m**a*	Mo *K*α radiation, λ = 0.71073 Å
Hall symbol: -P 2ac 2n	Cell parameters from 3391 reflections
*a* = 8.7128 (2) Å	θ = 3.6–27.5°
*b* = 12.7218 (4) Å	µ = 6.79 mm^−^^1^
*c* = 8.9377 (3) Å	*T* = 296 K
*V* = 990.68 (5) Å^3^	Platelet, colourless
*Z* = 4	0.22 × 0.15 × 0.11 mm

### Data collection

**Table:**

Bruker APEXII CCD area-detector diffractometer	1194 independent reflections
Radiation source: fine-focus sealed tube	1136 reflections with *I* > 2σ(*I*)
graphite	*R*_int_ = 0.019
Detector resolution: 8.3333 pixels mm^-1^	θ_max_ = 27.6°, θ_min_ = 2.8°
φ and ω scans	*h* = −7→11
Absorption correction: multi-scan (*SADABS*; Sheldrick, 2008a)	*k* = −16→16
*T*_min_ = 0.330, *T*_max_ = 0.460	*l* = −11→7
5338 measured reflections	

### Refinement

**Table:**

Refinement on *F*^2^	0 constraints
Least-squares matrix: full	Primary atom site location: structure-invariant direct methods
*R*[*F*^2^ > 2σ(*F*^2^)] = 0.030	Secondary atom site location: difference Fourier map
*wR*(*F*^2^) = 0.110	*w* = 1/[σ^2^(*F*_o_^2^) + (0.0553*P*)^2^ + 6.1916*P*] where *P* = (*F*_o_^2^ + 2*F*_c_^2^)/3
*S* = 1.22	(Δ/σ)_max_ < 0.001
1194 reflections	Δρ_max_ = 1.60 e Å^−^^3^
97 parameters	Δρ_min_ = −1.11 e Å^−^^3^
0 restraints	

### Special details

**Table:**

Geometry. All e.s.d.'s (except the e.s.d. in the dihedral angle between two l.s. planes) are estimated using the full covariance matrix. The cell e.s.d.'s are taken into account individually in the estimation of e.s.d.'s in distances, angles and torsion angles; correlations between e.s.d.'s in cell parameters are only used when they are defined by crystal symmetry. An approximate (isotropic) treatment of cell e.s.d.'s is used for estimating e.s.d.'s involving l.s. planes.
Refinement. Refinement of *F*^2^ against ALL reflections. The weighted *R*-factor *wR* and goodness of fit *S* are based on *F*^2^, conventional *R*-factors *R* are based on *F*, with *F* set to zero for negative *F*^2^. The threshold expression of *F*^2^ > σ(*F*^2^) is used only for calculating *R*-factors(gt) *etc*. and is not relevant to the choice of reflections for refinement. *R*-factors based on *F*^2^ are statistically about twice as large as those based on *F*, and *R*- factors based on ALL data will be even larger.

### Fractional atomic coordinates and isotropic or equivalent isotropic displacement parameters (Å^2^)

**Table:**

	*x*	*y*	*z*	*U*_iso_*/*U*_eq_	
Y1	0.01824 (6)	0.2500	0.05695 (6)	0.0042 (2)	
P1	0.49261 (13)	0.41750 (10)	−0.20198 (13)	0.0101 (3)	
P2	0.4300 (2)	0.2500	0.00645 (18)	0.0117 (4)	
P3	−0.24466 (12)	0.43269 (9)	0.22692 (13)	0.0104 (3)	
O1	0.2739 (6)	0.2500	−0.0586 (5)	0.0164 (10)	
O2	0.5265 (4)	0.3470 (3)	−0.0646 (4)	0.0148 (7)	
O3	−0.0382 (6)	0.2500	0.3332 (6)	0.0193 (11)	
O4	−0.1756 (4)	0.3732 (3)	0.1035 (4)	0.0166 (7)	
O5	0.6146 (4)	0.5051 (3)	−0.1804 (4)	0.0146 (7)	
O6	0.1567 (4)	0.3836 (3)	0.1598 (4)	0.0172 (7)	
O7	0.3374 (4)	0.4704 (3)	−0.1552 (4)	0.0150 (7)	
O8	−0.0066 (4)	0.3649 (3)	−0.1534 (4)	0.0191 (8)	

### Atomic displacement parameters (Å^2^)

**Table:**

	*U*^11^	*U*^22^	*U*^33^	*U*^12^	*U*^13^	*U*^23^
Y1	0.0044 (3)	0.0036 (3)	0.0045 (3)	0.000	−0.00084 (18)	0.000
P1	0.0105 (6)	0.0099 (6)	0.0101 (5)	−0.0001 (4)	0.0002 (4)	0.0000 (4)
P2	0.0129 (8)	0.0116 (8)	0.0107 (8)	0.000	0.0003 (6)	0.000
P3	0.0089 (5)	0.0100 (5)	0.0123 (5)	−0.0004 (4)	0.0002 (4)	−0.0001 (4)
O1	0.012 (2)	0.020 (2)	0.016 (2)	0.000	0.0002 (18)	0.000
O2	0.0141 (16)	0.0138 (17)	0.0165 (17)	−0.0023 (13)	−0.0028 (12)	0.0050 (13)
O3	0.020 (3)	0.022 (3)	0.015 (2)	0.000	0.001 (2)	0.000
O4	0.0158 (16)	0.0187 (17)	0.0154 (15)	0.0041 (14)	0.0008 (13)	−0.0021 (13)
O5	0.0139 (16)	0.0146 (15)	0.0153 (15)	−0.0052 (13)	0.0019 (13)	0.0009 (13)
O6	0.0153 (17)	0.0166 (16)	0.0196 (16)	−0.0025 (13)	−0.0035 (14)	−0.0032 (13)
O7	0.0125 (15)	0.0154 (15)	0.0172 (16)	0.0037 (13)	0.0021 (13)	0.0038 (13)
O8	0.0207 (19)	0.0215 (19)	0.0150 (16)	0.0009 (14)	0.0014 (13)	0.0058 (16)

### Geometric parameters (Å, °)

**Table:**

Y1—O6	2.278 (3)	P1—O5	1.553 (3)
Y1—O6^i^	2.278 (3)	P1—O7	1.567 (3)
Y1—O4^i^	2.341 (3)	P2—O3^iii^	1.460 (5)
Y1—O4	2.341 (3)	P2—O1	1.479 (5)
Y1—O8^i^	2.391 (4)	P2—O2	1.623 (4)
Y1—O8	2.391 (4)	P2—O2^i^	1.623 (4)
Y1—O1	2.455 (5)	P3—O4	1.467 (3)
Y1—O3	2.518 (5)	P3—O6^iv^	1.468 (3)
P1—O8^ii^	1.455 (4)	P3—O7^v^	1.607 (3)
P1—O2	1.549 (4)	P3—O5^vi^	1.611 (3)
			
O6—Y1—O6^i^	96.52 (18)	O1—Y1—O3	126.14 (16)
O6—Y1—O4^i^	144.08 (12)	O8^ii^—P1—O2	115.9 (2)
O6^i^—Y1—O4^i^	79.10 (13)	O8^ii^—P1—O5	115.9 (2)
O6—Y1—O4	79.10 (13)	O2—P1—O5	100.76 (19)
O6^i^—Y1—O4	144.08 (12)	O8^ii^—P1—O7	116.0 (2)
O4^i^—Y1—O4	84.04 (18)	O2—P1—O7	101.63 (19)
O6—Y1—O8^i^	145.21 (13)	O5—P1—O7	104.42 (19)
O6^i^—Y1—O8^i^	84.77 (13)	O3^iii^—P2—O1	124.1 (3)
O4^i^—Y1—O8^i^	70.44 (12)	O3^iii^—P2—O2	106.61 (19)
O4—Y1—O8^i^	118.93 (12)	O1—P2—O2	108.82 (17)
O6—Y1—O8	84.77 (13)	O3^iii^—P2—O2^i^	106.61 (19)
O6^i^—Y1—O8	145.21 (13)	O1—P2—O2^i^	108.82 (17)
O4^i^—Y1—O8	118.93 (12)	O2—P2—O2^i^	99.0 (3)
O4—Y1—O8	70.44 (12)	O4—P3—O6^iv^	122.6 (2)
O8^i^—Y1—O8	75.38 (19)	O4—P3—O7^v^	107.59 (18)
O6—Y1—O1	71.89 (11)	O6^iv^—P3—O7^v^	107.87 (19)
O6^i^—Y1—O1	71.89 (11)	O4—P3—O5^vi^	110.6 (2)
O4^i^—Y1—O1	136.82 (9)	O6^iv^—P3—O5^vi^	105.42 (19)
O4—Y1—O1	136.82 (9)	O7^v^—P3—O5^vi^	100.51 (18)
O8^i^—Y1—O1	75.61 (12)	P2—O1—Y1	132.0 (3)
O8—Y1—O1	75.61 (12)	P1—O2—P2	130.7 (2)
O6—Y1—O3	73.01 (12)	P2^iv^—O3—Y1	179.7 (3)
O6^i^—Y1—O3	73.01 (12)	P3—O4—Y1	140.8 (2)
O4^i^—Y1—O3	71.63 (12)	P1—O5—P3^vii^	140.4 (2)
O4—Y1—O3	71.63 (12)	P3^iii^—O6—Y1	155.0 (2)
O8^i^—Y1—O3	138.88 (10)	P1—O7—P3^v^	131.1 (2)
O8—Y1—O3	138.88 (10)	P1^viii^—O8—Y1	168.4 (3)

Symmetry codes: (i) *x*, −*y*+1/2, *z*; (ii) *x*+1/2, *y*, −*z*−1/2; (iii) *x*+1/2, *y*, −*z*+1/2; (iv) *x*−1/2, *y*, −*z*+1/2; (v) −*x*, −*y*+1, −*z*; (vi) −*x*+1/2, −*y*+1, *z*+1/2; (vii) −*x*+1/2, −*y*+1, *z*−1/2; (viii) *x*−1/2, *y*, −*z*−1/2.
